# An Analysis of Medical Students’ Attitude and Motivation in Pursuing an Intercalated MSc in Clinical Anatomy

**DOI:** 10.1007/s40670-019-00705-5

**Published:** 2019-02-12

**Authors:** Eiman Abdel Meguid, William E. Allen

**Affiliations:** Centre for Biomedical Sciences Education, School of Medicine, Dentistry & Biomedical Sciences, Queen’s University Belfast, 97 Lisburn Road, Belfast, Northern Ireland BT9 7AE UK

**Keywords:** Medical students, Project, Intercalated MSc, Clinical anatomy, Intercalated BSc

## Abstract

**Objectives:**

This study aimed to explore what factors influence and motivate medical students to undergo an intercalated degree and why they prefer to choose an intercalated MSc in Clinical Anatomy.

**Methods:**

The study consisted of 54 medical students enrolled in Queen’s University Belfast which offers a range of intercalated degrees, including an iBSc in Medical Science and an iMSc in Clinical Anatomy. Five-point Likert scale survey was used to collect data, designed to discover what the influencing factors were in deciding to take an intercalating degree and if they have a desire to gain research experience. It measured the motivational features of their chosen courses.

**Results:**

In recent years, more students (68.5%, *n* = 54) opted for the iMSc rather than the iBSc. This difference in number of students was statistically significant (chi-square = 33.4, *P* < 0.0001). It was theorized that this was due to an interest in future surgical specialization; however, this study has shown that the prime reason 72.2% of students opt to take a year out of their medical degree to carry out an intercalated degree is simply to gain an extra qualification whilst 61.1% thought it would enhance their competitiveness in the job market. Ninety-four percent of the iMSc students recommended the intercalated degree to junior students in comparison to only 34.8% of the iBSc students. This difference in percentage was statistically significant (*t* = 2.78, *P* = 0.009).

**Conclusion:**

The study shows no significant link to a desire to gain research experience in determining which intercalated programme to undertake. Students favoured iMSc more because they believed it will enhance their employability.

## Introduction

Undertaking an intercalated year whilst at medical school involves taking a year out of the medical degree in order to pursue a separate but related degree, followed by a subsequent return to the original course [1]. Typically, this year out of the undergraduate degree would involve an allocated period devoted to a research project.

Studies have shown that undertaking an intercalated degree is widely seen as a challenging but rewarding experience, with much to be gained from both the taught and the research components of the additional degree. Student’s opinion also reveals a positive attitude towards the intercalated degree, with those completing an additional degree showing a high level of satisfaction [2].

A number of factors may influence the choice of an intercalated degree, such as identification of mentors, finding research intellectually stimulating, future lifestyle and academic employment opportunities [3].

For those for whom medicine is their first degree, the novel stimuli and the breadth of knowledge required can be overwhelming. At medical school, universities aim to address this by instilling fundamental principles and by creating a firm foundation to prepare students for their future careers. However, whilst this might provide students with the core proficiencies necessary, it is often difficult to teach some of the independent skills essential for critically analyzing evidence in practice. Some skills are often best developed by undertaking research projects under the supervision of an experienced academic educator during an Intercalated Master (iMSc) or Bachelor’s (iBSc) degree. It is within this context that the issue of intercalation can be highlighted. Furthermore, the skills developed during an intercalated year are invaluable and help students prepare for taking a critical evidence-based approach to medicine. In addition, they are significantly more likely to take up positions in academia later in their career [4].

If a period of time is spent in research during the iMSc or iBSc, it will be beneficial for the medical students for a number of reasons [5, 6]. It may aid in the development of critical appraisal and problem-solving skills as well as lending an increased understanding of the process involved in developing surgical and laboratory research [5]. It will add a valuable period for reflection for the medical students [6].

The intercalated Bachelor of Science (iBSc) degree is one of the most common forms in which intercalation exists for medical students and has traditionally been held in high regard. It is reported that students who intercalate develop better deep and strategic learning skills compared with those who do not intercalate [1]. The literature suggests that those who have intercalated tend to do better academically, not only through the remainder of the medical school, but also after graduating [7, 8]. The pursuit of this degree is associated with better academic performance in surgical trainees and other programmes [9].

McManus and colleagues suggest that students who undertake an iBSc are better prepared for the rigours of academia and have an increased interest in pursuing research in their future careers. This is especially true in the United Kingdom (UK) where several medical schools already incorporate an iBSc or a Bachelor of Arts (BA) as a compulsory part of their undergraduate course, whilst the University of Nottingham integrates a similar BMedSci degree into their 5-year programme [1].

Many medical schools encourage intercalating, but, despite this, the issue of making intercalation mandatory is contentious, with counter-arguments ranging from the detrimental effect time taken out of the course can have to the lack of options available to cater for all students [10].

Benefits of intercalating should be presented to students in a way that helps them make a correct decision. It was noticed that over the past years, the number of medical students who have chosen to do an intercalated degree has been continuously decreasing despite the advantages in securing foundation posts [11]. In the UK and Ireland, data suggests that only a small percent of students graduating with an intercalated degree annually across all specialties go on to do surgical training; however, those that do, preserve an interest in laboratory-based academic surgery [12–14].

Queen’s University Belfast runs a unique (at the time of writing) iMSc in Clinical Anatomy alongside a MSc in Clinical Anatomy available to non-clinical graduates. The aims of this study were to analyze the attitude of intercalated medical students (enrolled in either the iBSc in Medical Science or iMSc in Clinical Anatomy programmes at the Queen’s University Belfast) and their motivation in pursuing an intercalated degree and to determine how important they found their engagement with a research project. This study explored the experience and motivation of students in carrying out the iMSc in Clinical Anatomy, and why iMSc in Clinical Anatomy students opted for this programme rather than the traditional iBSc. It investigated the influence of different factors on students when deciding to do an iMSc in Clinical Anatomy or the iBSc in Medical Science. The purpose of this article was also to provide an overview of the benefits of the iMSc in Clinical Anatomy to medical students and educators.

## Methods

### Participants

During the academic year 2016/2017, a total of 54 intercalated students—31 (45.6% of the total number of intercalating students) and 23 (33.8% of the total number)—were enrolled in the iMSc in Clinical Anatomy and iBSc in Medical Science, respectively, in the School of Medicine, Dentistry and Biomedical Sciences, Queen’s University Belfast (QUB), UK. They were invited to complete a paper-based five-point Likert scale survey.

The iBSc in Medical Science students were included in this research to study their attitudes, motivation, views and preferences with regard to the two intercalated programmes. After getting the approval of the Queen’s University Belfast, School of Medicine, Dentistry and Biomedical Sciences Research Ethics Committee (reference 16.64v3), the students completed a survey about their attitude and motivation in pursuing an intercalated degree towards the end of the academic year. The demographic breakdown of the medical students enrolled in iMSc in Clinical Anatomy was 77.4% females and 22.6% males whilst the iBSc in Medical Science students were 56.6% females and 43.5% males. In relation to their participation in the study, both anonymity and confidentiality were guaranteed.

### Educational Context

Intercalated students who participated in this study re-joined the medical curriculum again after the end of the 3rd year. The MB BCh BAO (Bachelor of Medicine) at the Queen’s University Belfast is a five-year programme.

#### iMSc in Clinical Anatomy Programme

Three modules are taught in one year during the iMSc, with a total of 48 h practical sessions and 24 h lectures for each module in addition to a research project that should be done in a period of 16 weeks. Specifically, this iMSc in Clinical Anatomy programme covers the structure and function of the trunk, limbs, back, head and neck and the central nervous system. Particular emphasis is made on the functional and clinical relevance of the three modules. This programme offers the opportunity for hands-on anatomy.

The Applied Anatomy of the Trunk module covers theoretical and practical aspects of applied clinical anatomy of the trunk, including the thorax, abdomen and pelvis. More specifically, it presents aspects of thoracic osteology; the cardiovascular and respiratory systems, including the vasculature and innervation, and the mediastinal structures, including relevant clinical aspects. It also covers the digestive system and biliary system, structural and functional relations of the peritoneum, together with the associated vasculature and innervation. The male and female pelvis and associated structures, vasculature and innervation and the structural and clinical aspects of the perineum are also studied.

The Applied Anatomy of the Limbs and Back module covers the detailed anatomy of the upper limb, lower limb and the back through the study of a range of human cadaveric specimens. Specifically, this includes the vasculature, innervation, musculature, osteology and joints of the limbs, together with aspects of the vertebral column osteology and arthrology; the intrinsic and extrinsic muscles of the back, including the appropriate vasculature, innervation and relevant clinical applications.

The Applied Anatomy of the Head and Neck region and the Central Nervous System module covers the detailed osteology of the cranium, face and cervical regions, including the oral, nasal and orbital cavities and muscles of the neck, face and mastication. The regional vasculature and its special clinical applications, lymph drainage of the head and neck region, the pharynx and laryngeal apparatus and the detailed neuroanatomy of the brain, spinal cord and cranial nerves are also studied.

Anatomy is traditionally delivered in the form of lectures and practical classes taught through dissection, prosected specimens, models and interactive small group activities to promote problem solving, to activate deeper on-going retention of information and to examine post-lecture knowledge recall. During dissection, a group of six students usually share a cadaver and dissect in a rotatory manner under the supervision of a lecturer. This lecturer, in addition, explains the steps of dissection participated in the discussion of clinically relevant information. Students were asked to identify anatomical structures, draw and label diagrams, examine models and solve clinical cases.

#### iBSc in Medical Science Programme

Students participating in this study were enrolled in the iBSc in Medical Science. The curriculum for iBSc in Medical Science includes a double module research project alongside two double taught modules from a choice of Embryology and Developmental Biology, Sports and Exercise Physiology, Advanced Neurosciences, Cardiovascular Pathobiology and treatment in addition to the Principles of Pharmacology and Therapeutics.

### Initial Data Collection

The primary research instrument was a structured paper-based survey to collect data and conduct the investigation. An information sheet about the research was given to the students along with the survey paper.

In section A of the survey, respondents scored nine items regarding the influence of some factors when deciding to do an intercalated degree. They were asked to answer a basic set of items related to their motivation to carry out an intercalated degree and the influence of certain factors when considering it.

In section B of the survey, twenty-eight items were a modified version of a previously validated and reliable instrument adapted from Keller [15] belonging to the instructional materials motivation survey (IMMS) that were designed to measure the motivational features of a course and attention, relevance, confidence and satisfaction (ARCS). The IMMS are based on Keller’s (ARCS) models of motivation, which purports to assess the motivational characteristics of courses or instructional materials using the Attention which is stimulated by deeper level of curiosity, Relevance that connects the content to important goals as academic requirements, Confidence that helps students establish positive expectancies for success and Satisfaction which is the positive feeling about the learning experience.

In this section, the items explored students’ experiences on carrying out the intercalated degree and how it affected their motivation. The ARCS model of motivation designed by Keller [15] provides guidance for analyzing the motivational characteristics of intercalated medical students for pursuing an intercalated degree and, then, for designing motivational strategies based on this analysis.

Both sections required students to answer by choosing one of the options using five-point Likert-scale survey ranging from “strongly disagree” to “strongly agree” by measuring subjective opinion on statements. Students were asked to rate the importance of the intercalated degree to their future career. The survey was designed in such a way as to ensure that the scale items suitably reflected the proposed domain.

### Data Analysis

Statistical analyses and interpretation of data were conducted using the SPSS statistical package, version 20 (IBM Corp, Armonk, NY). The analyses were done in a manner that prevents the identification of individuals. Results were statistically analyzed to explore the students’ attitude and motivation in pursuing an intercalated degree. Descriptive statistics, including frequency, distribution, mean and standard deviation, were calculated for each statement of the survey. Kolmogorov–Smirnov test was used to examine the normality of data distribution. The negatively framed questions were accounted for before starting the analysis. For the negatively framed statements, reverse mean was calculated during the analysis by using the following formula: reverse mean = 6 − normal mean.

Univariate analyses, including chi-square test, Monte Carlo test and Fisher’s exact test, were used to test the significance of results of qualitative variables. *t* test analyses were used to test the significance of results of quantitative variables and to compare the score differences of the background data, such as gender. The significance of the results was set at the 5% level of significance. A *P* value was considered significant if it is ≤ 0.05. *P* value was obtained using chi-square to test the significance of difference for every statement.

### Data Protection Issues

Prior to participation in this study, the students were informed that both anonymity and confidentiality were guaranteed. No specific information which may lead to the identification of an individual was released. Participants were made aware that the data were totally anonymous once it has been collected and that their participation was voluntary. Concerning the questionnaire, they were told that they could skip any question they did not want to answer.

The participants were aware of how the data were stored, and they were reassured that it was impossible for them to be identified. Computers used were password-protected. Data collected will be kept in a locked cabinet securely for at least 5 years after the research has been concluded. To alleviate the worries of the students, appropriate data security procedures and precautions were adopted so that data obtained were kept secured with access only by the researchers.

## Results

### Intercalated Programmes at Queen’s University Belfast

The Queen’s University Belfast (QUB) has for many years offered a selection of intercalated BSc programmes for medical students, with popular options being iBSc in Biochemistry and iBSc in Anatomy. In recent years, a growing number of intercalated postgraduate taught (PGT) programmes have been offered, including a MSc in Public Health and a MRes in Molecular Medicine. These PGT programmes had minimal impact on enrolment to the iBSc in Medical Science. However, the creation of the iMSc in Clinical Anatomy in 2015/16 has had a major impact on the number of students enrolled in the iBSc in Medical Science which was created earlier in 2012/2013 (Table 1).

**Table 1:** Intercalated degree enrollment at School of Medicine, Dentistry and Biomedical Sciences, Queen’s University Belfast

Year	Number of intercalating students	Number of iBSc in Medical Science students	% taking iBSc	Number of iMSc in Clinical Anatomy	% iMSc in Clinical Anatomy	Number of intercalated students in other iPGT	% taking other iPGT
2013/14	37	23	62	–	–	14	38
2014/15	33	21	63.6	–	–	12	36.4
2015/16	50	14	28	20	40	16	32
2016/17	68	23	33.8	31	45.6	14	20.6
2017/18	50	10	20	20	40	20	40
2018/19	48	5	10.4	34	70.8	9	18.8

Prior to the introduction of the iMSc in Clinical Anatomy, 37 QUB students opted to carry out an intercalating degree in 2013/14, of which 62% opted for the iBSc in Medical Science, with the remaining opting for a number of intercalating PGT programmes. Once the iMSc in Clinical Anatomy was introduced in 2015/2016, the number of students opting to intercalate increased by 26%. Despite this increase in numbers, the number of students who were enrolled in the iBSc in Medical Science declined by more than three quarters in 2018/2019, with the percentage of iBSc in Medical Science students dropped to be about 30% of the total number of intercalated students (Table 1).

There has been very little change in the number of students opting for PGT options other than the iMSc in Clinical Anatomy. This gave an indication that this programme is responsible both for the increased number of intercalating students and for the decline in iBSc enrolment (Table 1). In light of this observation, the authors of this study decided to investigate the attitude and experience of medical students who opted to carry out an intercalated degree in both iMSc in Clinical Anatomy and iBSc in Medical Science.

### Influencing Factors on Students When Deciding to Pursue an Intercalated Degree

The survey (Table 2) used to collect information about factors that influence students’ decisions to pursue an intercalated degree was completed by 54 intercalating students (31 iMSc in Clinical Anatomy and 23 iBSc in Medical Science), indicating a response rate of 100%. More students (68.5%) considered studying iMSc in Clinical Anatomy rather than the iBSc in Medical Science, and this difference was statistically significant (chi-square = 33.4, *P* < 0.0001). Only 13% of the iMSc in Clinical Anatomy students had a BSc either in Biomedical Sciences or in Human Biology before enrolling in this programme. The latter (8.84%) had a previous exposure to gross anatomy.

**Table 2:** Attitude of iBSc in Medical Science and iMSc in Clinical Anatomy students towards selecting an intercalated degree

Attitude and motivation								Significance
		iBSc (*n* = 23)		iMSc (*n* = 31)		Total (*n* = 54)		
		No.	%	No.	%	No.	%	
1—My motivation for carrying out an intercalated degree was the desire to gain research experience	SA/A	3	13.0	5	16.1	***8***	***14.8***	***X***^**2**^ **= 10.249** ^**MC**^***P*** = 0.004*
	Neutral	3	13.0	16	51.6	***19***	***35.2***	
	SD/D	17	74.0	10	32.3	***27***	***50.0***	
2—My motivation for carrying out an intercalated degree was the desire to get an extra qualification	SA/A	16	69.6	23	74.2	***39***	***72.2***	*X*^2^ = 1.387 ^**MC**^*P* = 0.623
	Neutral	1	4.3	0	0.0	***1***	***1.9***	
	SD/D	6	26.1	8	25.8	***14***	***25.9***	
3—An interest in a specific clinical specialty had influenced my decision to carry out an intercalated degree	SA/A	3	13.0	1	3.2	***4***	***7.4***	*X*^2^ = 2.245 ^**MC**^*P* = 0.330
	Neutral	4	17.4	4	12.9	***8***	***14.8***	
	SD/D	16	69.6	26	83.9	***42***	***77.8***	
4—Having an intercalated degree will enhance my competitiveness in the job market	SA/A	6	26.1	27	87.1	***33***	***61.1***	***X***^**2**^ **= 21.008** ^**MC**^***P*** < 0.0001*
	Neutral	2	8.7	0	0.0	***2***	***3.7***	
	SD/D	15	65.2	4	12.9	***19***	***35.2***	
5—The experience gained during an intercalated degree will make me a better medical doctor	SA/A	7	30.4	19	61.3	***26***	***48.1***	***X***^**2**^ **= 7.559** ^**MC**^***P*** = 0.016*
	Neutral	3	13.0	0	0.0	***3***	***5.6***	
	SD/D	13	56.6	12	38.7	***25***	***46.3***	
6—I selected modules which I felt would offer the most added benefit to my education	SA/A	18	78.3	31	100.0	***49***	***90.7***	***X***^**2**^ **= 7.427** ^**MC**^***P*** = 0.012*
	Neutral	2	8.7	0	0.0	***2***	***3.7***	
	SD/D	3	13.0	0	0.0	***3***	***5.6***	
7—I selected modules most aligned to modules in my medical degree	SA/A	13	56.5	24	77.4	***37***	***68.5***	*X*^2^ = 2.765 ^**MC**^*P* = 0.286
	Neutral	2	8.7	1	3.2	***3***	***5.6***	
	SD/D	8	34.8	6	19.4	***14***	***25.9***	
8—I expected the intercalated degree to be less demanding than undergraduate medical programme	SA/A	18	78.3	14	45.2	***32***	***59.3***	***X***^**2**^ **= 7.114** ^**MC**^***P*** = 0.030*
	Neutral	2	8.7	12	38.7	***14***	***25.9***	
	SD/D	3	13.0	5	16.1	***8***	***14.8***	
9—The greater cost of the iMSc Clinical Anatomy influenced my choice to enroll for an iBSc	SA/A	6	26.1	8	25.8	***14***	***25.9***	*X*^2^ = 4.181 ^MC^*P* = 0.124
	Neutral	8	34.8	18	58.1	***26***	***48.2***	
	SD/D	9	39.1	5	16.1	***14***	***25.9***	

SA/A, strongly agree/agree; SD/D, strongly disagree/disagree; *X*^2^, chi-square test; ^MC^P, Monte Carlo-corrected *P* value

*Significant at *P* ≤ 0.05

Seventy-four percent of the iBSc in Medical Science students strongly disagreed/disagreed that their motivation for carrying out an intercalated degree was the desire to gain a research experience compared to about 32.3% of the iMSc in Clinical Anatomy students with an observed statistical significance (*X*^2^ = 10.2496, ^MC^*P* = 0.004) between both cohorts of students. Eighty-seven percent (87.1%) of the iMSc in Clinical Anatomy students strongly agreed/agreed that having an intercalated degree would enhance their competitiveness in the job market in comparison to about a quarter (26.1%) of the iBSc in Medical Science students. This difference was statistically significant (*X*^2^ = 21.008, ^MC^*P* < 0.0001). All (100%) iMSc in Clinical Anatomy students strongly agreed/agreed that they selected modules, which they felt, would offer the most added benefit to their education compared to only 78.3% of the iBSc in Medical Science students. This difference was statistically significant (*X*^2^ = 7.427, ^MC^*P* = 0.012) (Table 2). Results showed that 77.4% of the iMSc in Clinical Anatomy students and 56.5% of the iBSc in Medical Science students strongly agreed/agreed that they selected modules that were most aligned to modules in their medical degree. This difference was statistically insignificant (*X*^2^ = 2.765, ^MC^*P* = 0.286) (Table 2).

It was also found that 78.3% of the iBSc in Medical Science students strongly agreed/agreed that they expected the intercalated degree to be less demanding than the undergraduate medical programme whilst only 45.2% of the iMSc in Clinical Anatomy students strongly agreed/agreed with this. This difference was statistically significant (*X*^2^ = 7.114, ^MC^*P* = 0.030) (Table 2).

### iBSc in Medical Science and iMSc in Clinical Anatomy Students’ Motivational Experience

In section B of the survey (Tables 3, 4, 5), twenty-eight items were a modified version of a previously validated and reliable instrument adapted from Keller [15] belonging to the instructional materials motivation survey (IMMS) that were designed to measure the motivational features of a course (ARCS). In this section, the items explored students’ experiences on carrying out the intercalated degree and how it affected their motivation.

**Table 3:** Perception of students towards items assessing relevance of their course

Relevance		Studied students						Significance
		iBSc (*n* = 23)		iMSc (*n* = 31)		Total (*n* = 54)		
		No.	%	No.	%	No.	%	
1—The intercalated degree research was applicable to my course of study	SA/A	3	13.0	8	25.8	11	20.4	*X*^2^ = 2.316 ^MC^*P* = 0.304
	Neutral	5	21.7	9	29.0	14	25.9	
	SD/D	15	65.3	14	45.2	29	53.7	
2—The knowledge I acquired during the intercalated degree will be useful to me in my future career	SA/A	19	82.6	31	100.0	50	92.6	***X***^**2**^ **= 5.823** ^**MC**^***P*** = 0.027*
	Neutral	2	8.7	0	0.0	2	3.7	
	SD/D	2	8.7	0	0.0	2	3.7	
3—During intercalated degree, I strived to achieve same high standard I wish to achieve in medical degree	SA/A	17	73.9	27	87.1	44	81.5	^FE^*P* = 0.294
	Neutral	0	0.0	0	0.0	0	0.0	
	SD/D	6	26.1	4	12.9	10	18.5	
4—I found the three years of the medical degree prepared me sufficiently for carrying out the intercalated degree	SA/A	21	91.4	29	93.5	50	92.5	***X***^**2**^ **= 1.460** ^**MC**^***P*** **= 0.754**
	Neutral	1	4.3	0	0.0	1	1.9	
	SD/D	1	4.3	2	6.5	3	5.6	
5—I struggled to adjust to the structure of the intercalated degree compared to the medical programme	SA/A	3	13.0	10	32.3	13	24.1	***X***^**2**^ **= 7.423** ^**MC**^***P*** = 0.019*
	Neutral	4	17.4	0	0.0	4	7.4	
	SD/D	16	69.6	21	67.7	37	68.5	
6—My experience of intercalated degree has increased my awareness of the importance of medical research	SA/A	20	87.0	23	74.2	43	79.6	*X*^2^ = 3.238 ^MC^*P* = 0.217
	Neutral	0	0.0	4	12.9	4	7.4	
	SD/D	3	13.0	4	12.9	7	13.0	
7—The content covered in intercalated degree allows me to understand my medical degree material better	SA/A	5	21.7	0	0.0	5	9.3	*X*^2^ = 7.821 ^MC^*P* = 0.017*
	Neutral	7	30.5	15	48.4	22	40.7	
	SD/D	11	47.8	16	51.6	27	50.0	
8—The intercalated experience has changed my approach to learning	SA/A	3	13.0	7	22.6	10	18.5	*X*^2^ = 0.855 ^MC^*P* = 0.682
	Neutral	3	13.0	3	9.7	6	11.1	
	SD/D	17	74.0	21	67.7	38	70.4	
9—The intercalated degree provided me with relevant knowledge	SA/A	21	91.3	27	87.0	48	88.9	*X*^2^ = 1.600 ^MC^*P* = 0.647
	Neutral	0	0.0	2	6.5	2	3.7	
	SD/D	2	8.7	2	6.5	4	7.4	

SA/A, strongly agree/agree; SD/D, strongly disagree/disagree; *X*^2^, chi-square test; MCP, Monte Carlo-corrected *P* value; FEP, Fisher’s exact test

*Significant at *P* ≤ 0.05

**Table 4:** Perception of students towards items assessing confidence

Confidence		Studied students						Significance
		iBSc (*n* = 23)		iMSc (*n* = 31)		Total (*n* = 54)		
		No.	%	No.	%	No.	%	
1—I am proud and excited about the outcome of my project	SA/A	4	17.4	2	6.5	6	11.1	*X*^2^ = 3.587 ^MC^*P* = 0.167
	Neutral	3	13.0	10	32.3	13	24.1	
	SD/D	16	69.6	19	61.2	35	64.8	
2—Information that had to be learned during the intercalated degree programme was just too difficult for me	SA/A	2	8.7	3	9.7	5	9.3	*X*^2^ = 0.059 ^MC^*P* = 1.0
	Neutral	1	4.3	1	3.2	2	3.7	
	SD/D	20	87.0	27	87.1	47	87.0	
3—I found the challenge of carrying out the project to be about right	SA/A	21	91.3	25	80.6	46	85.2	*X*^2^ = 1.870 ^MC^*P* = 0.510
	Neutral	2	8.7	4	12.9	6	11.1	
	SD/D	0	0.0	2	6.5	2	3.7	
4—During the intercalated degree, I had the impression that I would be successful in completing the tasks	SA/A	21	91.3	26	83.9	47	87.0	^FE^*P* = 0.685
	Neutral	2	8.7	5	16.1	7	13.0	
	SD/D	0	0.0	0	0.0	0	0.0	
5—It was difficult to predict how I would perform in the intercalated degree	SA/A	3	13.0	2	6.5	5	9.3	***X***^**2**^ **= 6.922** ^**MC**^***P*** = 0.028*
	Neutral	4	17.4	0	0.0	4	7.4	
	SD/D	16	69.6	29	93.5	45	83.3	
6—The research experience gave me confidence in achieving good marks in the intercalated degree	SA/A	5	21.7	5	16.1	10	18.5	***X***^**2**^ **= 14.757** ^**MC**^***P*** < 0.0001*
	Neutral	8	34.8	26	83.9	34	63.0	
	SD/D	10	43.5	0	0.0	10	18.5	

SA/A, strongly agree/agree; SD/D, strongly disagree/disagree; *X*^2^, chi-square test; MCP, Monte Carlo-corrected *P* value

*Significant at *P* ≤ 0.05

**Table 5:** Perception of students towards items assessing satisfaction and attention with regard to their course

Satisfaction & attention		Studied students						Significance
		iBSc (*n* = 23)		iMSc (*n* = 31)		Total (*n* = 54)		
		No.	%	No.	%	No.	%	
1—The learning outcomes for the intercalated degree were clear	SA/A	10	43.5	25	80.7	***35***	***64.8***	***X***^**2**^ **= 15.196** ^**MC**^***P*** = 0.001*
	Neutral	2	8.7	5	16.1	***7***	***13.0***	
	SD/D	11	47.8	1	3.2	***12***	***22.2***	
2—I found the material covered in the intercalated degree interesting	SA/A	18	78.3	24	77.4	***42***	***77.8***	*X*^2^ = 0.074 ^**MC**^*P* = 1.0
	Neutral	1	4.3	1	3.2	***2***	***3.7***	
	SD/D	4	17.4	6	19.4	***10***	***18.5***	
3—The effort to complete intercalated degree was appropriate for learning outcomes	SA/A	13	56.6	22	71.0	***35***	***64.8***	*X*^2^ = 1.268 ^**MC**^*P* = 0.609
	Neutral	5	21.7	4	12.9	***9***	***16.7***	
	SD/D	5	21.7	5	16.1	***10***	***18.5***	
4—I enjoyed the taught elements of the intercalated degree	SA/A	16	69.6	26	83.9	***42***	***77.8***	***X***^**2**^ **= 10.084** ^**MC**^***P*** = 0.005*
	Neutral	6	26.1	0	0.0	***6***	***11.1***	
	SD/D	1	4.3	5	16.1	***6***	***11.1***	
5—I enjoyed the research element of the intercalated degree	SA/A	0	0.0	2	6.5	***2***	***3.7***	*X*^2^ = 2.112 ^**MC**^*P* = 0.404
	Neutral	5	21.7	4	12.9	***9***	***16.7***	
	SD/D	18	78.3	25	80.6	***43***	***79.6***	
6—I would recommend the intercalated degree to junior medical students	SA/A	8	34.8	29	93.5	***37***	***68.5***	***X***^**2**^ **= 25.630** ^**MC**^***P*** < 0.0001*
	Neutral	1	4.3	2	6.5	***3***	***5.6***	
	SD/D	14	60.9	0	0.0	***14***	***25.9***	
7—I had to work hard to succeed in the intercalated degree	SA/A	16	69.6	26	83.8	***42***	***77.8***	*X*^2^ = 1.734 ^**MC**^*P* = 0.543
	Neutral	2	8.7	2	6.5	***4***	***7.4***	
	SD/D	5	21.7	3	9.7	***8***	***14.8***	
8—My experience of intercalated degree makes it more likely that I will pursue a career in academic medicine	SA/A	2	8.7	2	6.5	***4***	***7.4***	*X*^2^ = 0.117 ^**MC**^*P* = 1.0
	Neutral	4	17.4	6	19.4	***10***	***18.5***	
	SD/D	17	73.9	23	74.1	***40***	***74.1***	
9—Completing the research work gave me a satisfactory feeling of accomplishment	SA/A	1	4.3	2	6.5	***3***	***5.6***	***X***^**2**^ **= 19.683** ^**MC**^***P*** < 0.0001*
	Neutral	1	4.3	19	61.2	***20***	***37.0***	
	SD/D	21	91.4	10	32.3	***31***	***57.4***	
10—I am satisfied with the project’s results of my intercalated degree	SA/A	23	100.0	20	64.5	***43***	***79.6***	^**FE**^***P*** = 0.001*
	Neutral	0	0.0	11	35.5	***11***	***20.4***	
	SD/D	0	0.0	0	0.0	***0***	***0.0***	
11—The intercalated degree curriculum met my learning expectations and goals	SA/A	19	82.6	30	96.8	***49***	***90.7***	^FE^*P* = 0.151
	Neutral	0	0.0	0	0.0	***0***	***0.0***	
	SD/D	4	17.4	1	3.2	***5***	***9.3***	
12—The intercalated degree had a lot that captured my attention	SA/A	23	100.0	26	83.9	***49***	***90.7***	^**FE**^*P* = 0.064
	Neutral	0	0.0	5	16.1	***5***	***9.3***	
	SD/D	0	0.0	0	0.0	***0***	***0.0***	
13—The amount of effort during the intercalated degree caused me to get bored sometimes	SA/A	6	26.1	5	16.1	***11***	***20.4***	*X*^2^ = 1.141 ^**MC**^*P* = 0.612
	Neutral	6	26.1	7	22.6	***13***	***24.0***	
	SD/D	11	47.8	19	61.3	***30***	***55.6***	
14—My curiosity was often stimulated during the laboratory work in the project	SA/A	s20	87.0	31	100.0	51	94.4	*X*^2^ = 4.281 ^MC^*P* = 0.072
	Neutral	2	8.7	0	0.0	2	3.7	
	SD/D	1	4.3	0	0.0	1	1.9	

SA/A, strongly agree/agree; SD/D, strongly disagree/disagree; *X*^2^, chi-square test; ^MC^P, Monte Carlo-corrected *P* value; ^FE^P, Fisher’s exact test

*Significant at *P* ≤ 0.05

The relevance of the programme to the course was assessed by nine items (Table 3). All students (100%) strongly agreed/agreed that the knowledge they acquired during the iMSc in Clinical Anatomy degree would be useful in their future career in comparison to only 82.6% of the iBSc in Medical Science students. This difference was statistically significant (*X*^2^ = 5.823, ^MC^*P* = 0.001). The percentage of the iMSc in Clinical Anatomy students who strongly agreed/agreed about the 9 items that assessed relevance in Table 3 were higher than the iBSc in Medical Science students with regard to 5 out of 9 items.

An overwhelming majority of students of the iBSc in Medical Science (91.4%) and iMSc in Clinical Anatomy (93.5%) strongly agreed/agreed that the three years of the medical degree programme prepared them sufficiently for carrying out the intercalated degree. There was no statistical difference between the two cohorts of students. Only 13% of the iBSc in Medical Science students strongly agreed/agreed that they struggled to adjust to the structure of the intercalated degree compared to the medical programme, whilst the percentage of the iMSc in Clinical Anatomy students who strongly agreed/agreed was almost triple (32.3%). This difference was statistically significant (*X*^2^ = 7.423, ^MC^*P* = 0.019). More than two thirds of the iBSc in Medical Science (87%) and iMSc in Clinical Anatomy (74.2%) students strongly agreed/agreed that their experience of an intercalated degree has increased their awareness of the importance of the medical research. No statistical significance was noted between these two cohorts of students (*X*^2^ = 3.238, ^MC^*P* = 0.217) (Table 3).

Six items in the survey assessed students’ confidence. There was a statistically significant difference between the two cohorts of students in two of these items. Only 13% of the iBSc in Medical Science students and 6.5% of the iMSc in Clinical Anatomy students strongly agreed/agreed that it was difficult to predict how they would perform in the intercalated degree. This difference was statistically significant (*X*^2^ = 6.922, ^MC^*P* = 0.028). Twenty-one percent (21.7%) of the iBSc in Medical Science students and 16.1% of the iMSc in Clinical Anatomy students strongly agreed/agreed that the research experience gave them confidence in achieving good marks in the intercalated degree. This difference was statistically significant (*X*^2^ = 14.757, ^MC^*P* = 0.0001) (Table 4).

Students’ satisfaction was assessed using eleven items in Table 5 (1–11), whilst three items assessed their attention (12–14). Students enrolled in the iMSc in Clinical Anatomy were more satisfied with their programme than the students enrolled in the iBSc in Medical Science. The percentage of the iMSc in Clinical Anatomy students who strongly agreed/agreed about items (1–11) that tested satisfaction was higher than in the iBSc in Medical Science in most of them. This difference was found to be statistically significant in 5 out of 11 items (Table 5). Only 43.5% of the iBSc in Medical Science students strongly agreed/agreed that the learning outcomes for the intercalated degree were clear, whilst 80.7% of the iMSc in Clinical Anatomy students had the same opinion (*X*^2^ = 15.196, ^MC^*P* = 0.001) (Table 5). Eighty-four percent (83.9%) of the iMSc in Clinical Anatomy students strongly agreed/agreed that they enjoyed the taught elements, whilst only 69.6% of the iBSc in Medical Science students were found to strongly agree/agree about this. This difference was statistically significant (*X*^2^ = 10.084, ^MC^*P* = 0.005*). An overwhelming majority (93.5%) of the iMSc in Clinical Anatomy students would recommend the intercalated degree to junior medical students in comparison to only 34.8% of the iBSc in Medical Science students (*X*^2^ = 25.630, ^MC^*P* < 0.0001*) (Table 5). This overall difference was statistically significant (*t* test = 2.78, *P* = 0.009). The results of the statistical analysis using *t* test to explore the differences between students with regard to their gender identified that female students were more confident about their learning, and this difference was statistically significant (*t* test = 2.20, *P* = 0.032).

With regard to assessing attention in Table 5, two out of three items (12–14) were in favour of the iMSc in Clinical Anatomy programme.

## Discussion

One third of medical students in the UK pursued an intercalated degree in the previous years. These numbers were found to be declining [11]. Students have most commonly reported that their decision not to intercalate was due to financial obstacles or a lack of interest in research due to inadequate exposure to medical research [16]. A lack of exposure to proper medical research is something that can be addressed throughout universities in the UK.

The small numbers of students who decided to intercalate in Aberdeen in the academic year 2008/2009 was just under 18%, and, at Queen’s University Belfast, at the academic year 2016/2017, it was almost the same (21%).

It is clear that students are unaware of the benefits of intercalating degree, such as the benefits in terms of gaining an extra bonus in the academic ranking system for applications for foundation year posts and better performance in years 4 and 5 of their MBChB as observed by Cleland and colleagues [17]. Improved performances will further lead to better academic ranking in their career later on.

### Students’ Motivations for Taking an Intercalated Degree

The most striking result for the items that probed why students opted to take a year out of medicine to study for an intercalated degree for 69.6% of those who picked the iBSc in Medical Science and 74.2% of those who picked the iMSc in Clinical Anatomy was getting an extra qualification (Table 2). Interestingly, 87.1% of the iMSc in Clinical Anatomy students believe that an intercalated degree will enhance their employability in comparison to only 26.1% of those who opted for iBSc in Medical Sciences. This coincided with the findings of Stubbs and colleagues who found that the most common reason for 69.6% of students in deciding to take an intercalated degree was that it may help in getting the job they want [2]. Other than the desire to gain an extra qualification, those who opted for the iBSc in Medical Science have no particular educational or training motivation, whilst 61.3% of those who have opted for the iMSc in Clinical Anatomy believe that the degree will enhance their ability as medical practitioners. Disappointingly, most students in the two cohorts indicate little interest in gaining research experience as a motivation for intercalating as only 13% of the iBSc in Medical Science cohort and 16.1% of the iMSc in Clinical Anatomy cohort responded positively for this item.

The uptake of an academic career among medical students is undeniably a complex and dynamic process, shaped by the cumulative effect of past experiences and related behavioural domains [18]. Shah and colleagues [18] found that the most significant barriers to conducting research by students were “lack of time” (72.4%) and “lack of good supervisors” (50.3%). Students would be more interested in research if more support and guidance is provided. Although research work is a heavy workload, researchers are not very well paid compared to clinicians. Shah and colleagues [18] noted that despite promising trends in research knowledge and practices, students’ future intentions to pursue medical research professionally remained low. Effective uptake of research among medical students must be guided by a theoretically informed approach comprising of capacity building for research-naïve students [18].

Findings of another study showed that lack of students’ professional statistical education made them face problems in research work [19]. Lack of sufficient numbers of experienced and keen supervisors who can act as guide plays a role in diminishing students’ interest in research. This finding corresponds with the data provided by Shirbagi [20].

There was a near universal accord regarding the recognition of the decline and shortage of physician-scientists as a problem that demanded serious attention. The literature was replete with expressed concerns about the lack of enthusiasm on the part of budding physicians to pursue the physician-scientist career track. Indeed, although many medical students indicated the importance of research in their education, they were not favourably inclined to pursue it as a career [21]. Various reasons were adduced for the lack of interest in a clinical research career path. Some of those reasons were preference for direct patient care as a clinician, lifestyle of a physician rather than a physician-scientist, diminished desire in prolonged training and accrual of extra debt, need to pay off current student debt, difficulty in seeking extra-mural funds, lack of mentorship and lack of interest in a scientific/research career [21, 22].

It is also surprising that those who opted for the iMSc in Clinical Anatomy did not choose intercalation because they are interested in surgery or any particular clinical specialty as only 3.2% agreed with this statement.

### Students’ Satisfaction with iBSc in Medical Science and iMSc in Clinical Anatomy Programmes

The study also aimed to better understand the level of satisfaction both cohorts of students feel regarding their experience of intercalating, as changes in programme selection could be influenced by word-of-mouth from one-year group to the next. For example, would iMSc in Clinical Anatomy students find their degree more satisfying than those who had opted for the iBSc in Medical Sciences?

The results indicated that those who opted for the iMSc in Clinical Anatomy were more satisfied with their experience than those who took the iBSc in Medical Sciences. Of particular relevance, 93.5% of the iMSc in Clinical Anatomy students are more likely to recommend the programme to following year groups and that was three times more than those who opted for the iBSc in Medical Sciences, as only 34.8% of them agreed with this statement (Table 2). This, therefore, could be a major factor in the continued popularity of the iMSc in Clinical Anatomy and decline in popularity for the iBSc in Medical Science. These findings are in line with the results of Stubbs and colleagues who found that a big percentage of intercalating students reported that they have gained an additional advantage over their classmates as well as extra skills that would be useful to their future careers [2]. Collins and colleagues stated that their intercalated students were satisfied with their performance in the year as a whole and with the help they received from their supervisors [23].

The majority of both groups of students (82.6% of iBSc in Medical Science and 100% of iMSc in Clinical Anatomy) felt that what they have been taught will be useful in their future medical career. However, they felt that it does not actually help them to understand their core medical knowledge as only 21.7% of iBSc in Medical Science and 0% of iMSc in Clinical Anatomy students agreed with the statement, “The content covered in intercalated degree allows me to understand my medical degree material better” (Table 3). Most did not feel that the research element was likely to be useful as only 21.7% of iBSc in Medical Science and 16.1% of iMSc in Clinical Anatomy students agreed with the statement, “The research experience gave me confidence in achieving good marks in the intercalated degree”. They did feel that the programmes as a whole enhanced their appreciation for the importance of medical research as 87% of iBSc in Medical Science and 74.2% of iMSc in Clinical Anatomy students agreed with the statement, “My experience of intercalated degree has increased my awareness of the importance of the medical research” (Table 3, Table 4). Research activity and publications benefit not only the future career of students but also their schools as medical intercalated student publications can significantly increase the research output of the faculty [24].

### Discussion of the Outcomes of This Study

It is to be assumed that if more intercalating medical students were supervised by more clinical academics/clinicians, the research would be more effective in terms of clinical applications and additional clinical training, which would benefit their future career and make students more appreciative to the research project. This opinion is consistent with that of Stubbs and colleagues who stated that intercalated students gained significantly more presentation experience and publications [2]. They showed a trend to have more first-class honors. Statistical analysis and in-depth research skills are among the transferable skills that can be acquired from an intercalated degree (both scientific and literature based) [2].

In this study, students clearly value intercalated degrees. This finding coincided with that of Stubbs and colleagues [2]. However, they mentioned that the rise in tuition fees would decrease the number of medical students who would choose an intercalated degree which would contribute to the further reduction in the number of graduates that would select an academic career. This agreed with the findings of McManus and colleagues [1]. In contrast to that, the current study found that only 26.1% of iBSc in Medical Science students and 25.8% of iMSc in Clinical Anatomy students agreed with the statement, “The greater cost of the iMSc Clinical Anatomy influenced my choice to enrol for an iBSc”.

Items in the survey aimed at accessing the levels of confidence that the two groups of students felt regarding their ability to carry out the research element of the programme. These items showed an ambivalent attitude regarding this key aspect of the programme with 91.3% of iBSc in Medical Science students and 80.6% of iMSc in Clinical Anatomy students agreeing with the statement, “I found the challenge in carrying out the project to be about right”. However, only 21.7% of iBSc in Medical Science students and 16.1% of iMSc in Clinical Anatomy students agreed with the statement, “The research experience gave me confidence in achieving good marks in the intercalated degree”.

Cleland and colleagues and Evered and colleagues stated that intercalated degrees would contribute to short-term benefits to students, such as better exam performance in the fourth year and the ability of having better strategic learning. Intercalating students are also likely to have published papers in academic journals [17, 25]. The combination of better examination grades, an intercalated degree qualification and a published paper or abstract increases their chances of getting their preferred choice of foundation school and rotation [17].

According to Blundell and colleagues and Cleland and colleagues, the reasons for not intercalating include the perception of the student body, such as lack of awareness or misunderstanding, financial considerations and time-costs or a lack of interest in undertaking research [17, 26]. Intercalated students would also worry about falling behind their peers in the progress of their careers [27].

Medical student intake in the UK has increased by 60% since 1997; yet, the body of clinical academics—those charged with designing, developing and implementing the curriculum—has shrunk by over 12%, since 2001 [28]. Intercalated degrees, which allow medical students the opportunity to conduct in-depth research themselves, may be part of the solution by introducing students to academic careers at an early stage in their training [29].

### Advantages of Being Enrolled in iMSc in Clinical Anatomy

By introducing an optional iMSc in Clinical Anatomy, including a research project, we may help to safeguard the next generation of academic surgeons, as trainees will be well equipped with the requisite research knowledge and skills to facilitate career-long interaction with the research community. If candidates have been furnished with their research skills and anatomical knowledge, they should be able to progress through streamlined “run-through” programmes without having to take time out. However, in the context of the challenges facing surgical training, the authors of the current study believe an iMSc in Clinical Anatomy for medical students may offer one strategy to encourage the next generation of academic surgeons.

## Conclusion

In *conclusion*, this study has indicated that the prime reason students opt to take a year out of their medical degree to carry out an intercalated degree is simply to gain an extra qualification to enhance their employability. The study shows no significant link to a desire to gain research experience or even to gain advantage for a future specialization. This indicates that more effort should be made by the medical school to inform medical students why and how each particular intercalated programme could benefit them beyond simply having an extra degree. Intercalated degrees would help the doctors of tomorrow to be better equipped to practice medicine in disciplines that are constantly evolving [30].

There are limitations to the current study; the first of these is the non-randomized selection of study participants from a single centre, which limits generalizability. A small-scale, exploratory study was conducted with students enrolled in two different intercalated degree programmes (iBSc in Medical Science and iMSc in Clinical Anatomy). A qualitative approach using focus group interviews would have added more information. The rest of the intercalated programmes, such as MSc in Public Health, MSc in Bioinformatics and Computational Genomics, MSc in Molecular Pathology of Cancer, MSc in Experimental Medicine, BSc in Biochemistry, BSc in Microbiology and BSc in Intercalated Psychology, were not included in this study due to the small number of students enrolled in these programmes. However, the authors are confident that these different intercalated programmes are important in developing our next generation to be either academics or clinicians. Formal assessment of the programme, to assess short-term and long-term outcomes of intercalation, was not carried out.

The findings of this study show that students need to be better made aware of the value of an intercalated degree in preparing for their future developments in research and practice and for their life-long learning and professional development.
